# Variably Sized and Multi-Colored Silica-Nanoparticles Characterized by Fluorescence Correlation Methods for Cellular Dynamics

**DOI:** 10.3390/ma14010019

**Published:** 2020-12-23

**Authors:** Chan-Gi Pack, Bjorn Paulson, Yeonhee Shin, Min Kyo Jung, Jun Sung Kim, Jun Ki Kim

**Affiliations:** 1 Asan Institute for Life Science, Asan Medical Center, Seoul 05505, Korea; changipack@amc.seoul.kr (C.-G.P.); bjorn.paulson+mtrls@gmail.com (B.P.); 2 Department of Convergence Medicine, University of Ulsan College of Medicine, Seoul 05505, Korea; dusgo147@gmail.com; 3 Neural Circuits Group, Korea Brain Research Institute, Daegu 41062, Korea; jungminkyo1@gmail.com; 4 Research and Development Center, H-MED Incorporated, Seoul 03761, Korea

**Keywords:** fluorescence correlation method, silica-based fluorescent nanoparticle, TEM, dual-colored nanoparticle, cobalt ferrite silica nanoparticle, hydrodynamic diameter, endocytosis

## Abstract

Controlling the uptake of nanoparticles into cells so as to balance therapeutic effects with toxicity is an essential unsolved problem in the development of nanomedicine technologies. From this point of view, it is useful to use standard nanoparticles to quantitatively evaluate the physical properties of the nanoparticles in solution and in cells, and to analyze the intracellular dynamic motion and distribution of these nanoparticles at a single-particle level. In this study, standard nanoparticles are developed based on a variant silica-based nanoparticle incorporating fluorescein isothiocyanate (FITC) or/and rhodamine B isothiocyanate (RITC) with a variety of accessible diameters and a matching fluorescent cobalt ferrite core-shell structure (Fe_2_O_4_/SiO_2_). The physical and optical properties of the nanoparticles in vitro are fully evaluated with the complementary methods of dynamic light scattering, electron microscopy, and two fluorescence correlation methods. In addition, cell uptake of dual-colored and core/shell nanoparticles via endocytosis in live HeLa cells is detected by fluorescence correlation spectroscopy and electron microscopy, indicating the suitability of the nanoparticles as standards for further studies of intracellular dynamics with multi-modal methods.

## 1. Introduction

A comprehensive understanding of the interplay between nanoparticles (NPs) and cellular organelles is essential to the development of nanotechnology-based diagnosis and therapies. Despite a presently limited understanding of NP uptake, NPs as drug-carriers have been applied for a myriad of uptake and cellular functions due to their controllable physicochemical characteristics [1,2,3]. Nanoparticle-based treatments have been proposed for radiation sensitization [4]; for photodynamic and photothermal therapy [5]; for the targeted delivery of drugs [6]; and for the detection of DNA, proteins, and bacteria [7], among many other applications. On this background, there is immense interest in the manner in which NPs interact with living systems, which is necessary for the development of safe and reliable therapeutic nanomedicine [3,8,9,10].

Fluorescently labelled NPs are primary tools for the investigation of nanoparticle interactions with biological systems at subcellular scales. Among them, silica-based NPs are particularly desirable for fluorescence modification, as fluorophores incorporated into NPs demonstrate higher brightness and photostability [11], while silica NPs facilitate surface modification and exhibit low cytotoxicity [12,13,14,15,16]. Incorporation of multiple fluorophores into single nanoparticles, such as combinations of fluorescein isothiocyanate (FITC) and rhodamine B isothiocyanate (RITC) into mesoporous silica nanoparticles, is known to result in Förster resonance energy transfer (FRET). Via FRET, a plethora of possible fluorescence spectra may be extracted from a single excitation wavelength [17], and controllable sensitivity to environmental conditions may be designed. The utility of FITC and RITC co-labelled NPs for cellular localization has been vividly demonstrated in the form of three-channel fluorescence microscopy of stained cells incorporating dual-fluorophore core/shell NPs [18]. Further surface modification of NPs, along with tailoring of their size and shape, allows control over NP uptake, cellular persistence, and cellular toxicity, and thus, control over their cellular fate.

However, the cellular behavior of synthesized NPs can be still challenging to control and even more challenging to measure. Uptake of NPs into cells is known to depend upon size, shape, and surface charge [19,20,21]. In analyzing the interactions between NPs and cells, confocal laser scanning microscopy (CLSM) can image the behavior of NPs in living cells, but is limited in its ability to accurately define the NP size. In contrast, electron microscopy (EM) can confirm the size and distribution of ultra-fine structures at the nano-scale, while providing information specific to fixed cells or dried samples. In biological solutions, NPs adsorb a corona of proteins or small molecules in a pH-dependent manner, resulting in a larger effective diameter (the hydrodynamic diameter) and different cellular entry conditions. In order to characterize nanomaterials and overcome these difficulties, myriad optical techniques have been developed [22] including dynamic light scattering (DLS), nanoparticle tracking analysis (NTA), fluorescence correlation spectroscopy (FCS), and dual-colored fluorescence cross-correlation spectroscopy (FCCS), among others. Each of these techniques has different advantages and disadvantages for applicability. These methods are commonly used to determine hydrodynamic diameter of NPs. Unlike other techniques, FCS and FCCS are naturally combined with CLSM, allowing the size characteristics and diffusion behavior of fluorescent NPs distributed in living cells and in solution samples to be detected at the level of single particles [15,23]. In addition to particle size measurement, FCS is also a useful technique for quantifying photostability and fluorescence brightness of fluorescent NPs, while FCCS has been a tool for the monitoring of intracellular interactions, providing access to binding interactions between fluorescent molecules as well as to enzymatic reactions [23].

Among nanoparticles, silica NPs are highly versatile, being highly non-toxic, allowing easy surface functionalization, and easily incorporating high dye payloads [24]. Several different methods have been proposed for the synthesis of core/shell NPs incorporating fluorescent silica over a cobalt ferrite core [25,26,27]. While ferrous silica NPs have been applied to myriad biomedical applications, including bacterial detection [28], tumor detection [29,30,31], and intracellular sensing [7], analyses of the cellular uptake process of cobalt-based core/shell NPs in single living cells have been almost exclusively limited to tumor treatment [32,33,34]. In order to understand how these various NPs enter the cell, diffuse, and interact, it will be important to analyze them using standard NPs with well-defined physicochemical properties.

In this paper, a novel synthesis of non-ferrous silica-based nanoparticles (SiO_2_-NPs) and CoFe_2_O_4_/SiO_2_ core-shell nanoparticles (CoFe_2_O_4_/SiO_2_-NPs) is presented, which provides NPs suitable for fluorescence correlation and EM studies of cellular NP uptake and of intracellular NP dynamics. The NPs incorporate the common fluorescent dyes of green FITC and red RITC into silica-based NPs with diameters ranging from 22 to 226 nm in diameter. A cobalt-iron-oxide core can be incorporated into the NPs, making them suitable for multi-modal studies that combine transmission electron microscopy (TEM), fluorescence correlation spectroscopy, and fluorescence cross-correlation spectroscopy. The ratio of fluorophores in the NPs can be controlled at the time of synthesis, resulting in a variety of fluorescent and energy-transfer behavior, which may distinguish different batches of NPs. Diameter and hydrodynamic properties of the synthesized NPs are quantified in aqueous solution and with dried samples by DLS, FCS and EM. Fluorescence cross-correlation spectroscopic detection of dual-colored NPs during endocytosis is preliminarily demonstrated on HeLa cells. While much work remains to quantify the strengths and biocompatibility of these NPs, their synthesis and analytical application for cell uptake processes promises to support multi-modal imaging for improving understanding of cellular uptake and interaction processes in live cells.

## 2. Materials and Methods

All commercially available chemical reagents were used without further purification: FeCl_3_·6H_2_O, CoCl_2_·6H_2_O, Fe(NO_3_)_3_·9H_2_O (Sigma-Aldrich, St. Louis, MO, USA), rhodamine B isothiocyanate and fluorescein isothiocyanate (Sigma-Aldrich), 3-aminopropyltriethoxysilane (Gelest, Morrisville, PA, USA), tetraethyl orthosilicate (Sigma-Aldrich), 2-(methoxy[polyethyleneoxy]propyl) trimethoxysilane (PEG-Si(OMe)_3_; Gelest), and polyvinylpyrrolidone (M_w_ 55,000; Sigma-Aldrich).

### 2.1. Synthesis of Fluorescent Silica-Based Nanoparticles

A 0.0694 g quantity of 3-aminopropyltriethoxysilane (APS; 0.03 mmol) was added to 0.0835 g of rhodamine B isothiocyanate (RITC) or/and fluorescein isothiocyanate (FITC) in 5 mL of anhydrous ethanol. This mixture was stirred in the dark at room temperature for 17 h, and was used as the APS-conjugated dye solution in the next step. To produce the silica-based fluorescent nanoparticles, tetraethyl orthosilicate (TEOS; 0.75 mL, 3.6 mmol) and the APS-conjugated dye solution were mixed with 42.7 mL of ethanol, and then 2.3 mL (4.6 vol%) of ammonium hydroxide solution (29% in H_2_O) was added. The total volume of the solution was adjusted to 50 mL. This reaction mixture was shaken at 25 °C for a time proportionate to the desired particle size. After the reaction, the nanoparticles (SiO_2_-NPs) were isolated by centrifugation (20,000× *g*, 20 min) and washed twice with ethanol. In the washing process, the precipitated particles were redispersed by sonication. Finally, the nanoparticles were resuspended in PBS solution to 2 mg mL^−1^.

The synthesis of CoFe_2_O_4_/SiO_2_ (RITC or FITC): Cobalt ferrite solution (34.7 mL; 20 mg mL^−1^ in H_2_O) was prepared by adding to polyvinylpyrrolidone (PVP) solution (0.65 mL; Mr 55,000 Da, 25.6 g L^−1^ in H_2_O), and the mixture was stirred for 24 h at room temperature. By addition of aqueous acetone (H_2_O/acetone = 1/10, *v*/*v*), the PVP-stabilized cobalt ferrite nanoparticles were separated and then centrifuged at 4000 rpm for 10 min. The supernatant solution was removed, and the precipitated particles were redispersed in ethanol (10 mL). Rhodamine B isothiocyanate and APS under N_2_ were prepared by Schlenck line into RITC-modified trimethoxysilane (RMT). A solution of TEOS and RMT was mixed at molar ratio 0.3 TEOS: 0.04 RMT and injected into the solution of PVP-stabilized cobalt ferrite in ethanol. Adding ammonia solution (0.86 mL; 30 wt % by NH_3_) as a catalyst initiated polymerization, producing cobalt ferrite–silica core–shell nanoparticles containing FITC or RITC, which were dispersed in ethanol and isolated by ultra-centrifugation (18,000 rpm, 30 min). The purified core–shell nanoparticles were redispersed in solvent for measurement [26,35,36].

### 2.2. Cell Culture and Uptake

Human epithelial carcinoma (HeLa) lines were obtained from the Korean Cell Line Bank (KCLB) (Seoul, Korea). HeLa cells were grown in a 5% CO_2_ humidified atmosphere at 37 °C in Dulbecco’s modified Eagle’s medium (DMEM; Sigma-Aldrich) supplemented with 10% FBS, 100 U mL^−1^ penicillin, and 10 mg mL^−1^ streptomycin. The cells were cultured in an eight-well chambered coverglass (Nunc, Roskilde, Denmark) to allow live cell imaging and FCS measurements.

### 2.3. Transmission Electron Microscopy

The silica-based fluorescent NPs diluted in ethanol were deposited on a copper grid (300 mesh, covered with carbon), and their images were taken with a JEM 30101 (JEOL, Tokyo, Japan) transmission electron microscope at 300 kV. For fixed cell sample assays, cells were incubated with test solutions, and after 2 h, the culture plates were fixed for 30 min in 2.5% glutaraldehyde in 0.1 M sodium cacodylate buffer (pH 7.4), fixed in 2% OsO4 for 1 h, and stained overnight en bloc in 2% uranyl acetate in distilled water. The cells were then dehydrated in a series of ethanol concentrations (50%, 60%, 70%, 80%, 90%, 95%, and 100%) for 20 min each and embedded in Spurr’s resin. After the resin hardened, the slide was removed from the block. An ultramicrotome (MTX-L, RMC, Boeckeler Instruments Inc., Tucson, AZ, USA) was used to prepare ultrathin sections cut horizontally to the bottom of the slide at a nominal thickness of 60 nm. Sections were stained with 2% uranyl acetate in 50% methanol followed by lead citrate and observed using a Tecnai 12 electron microscope operated at 120 kV (FEI, Hillsboro, OR, USA).

### 2.4. Dynamic Light Scattering and Zeta Potential Measurements

The hydrodynamic diameters and surface charges of the silica-based/core-shell nanoparticles were measured using the ELS-8000 electrophoretic light scattering apparatus (Photal, Osaka, Japan). The particles were washed with borate buffer (pH 9.0) and measured in distilled H_2_O.

### 2.5. Fluorescence Correlation Spectroscopy and Dual-Color Fluorescence Cross-Correlation Spectroscopy

FCS and FCCS measurement of fluorescent nanoparticles were performed using a ConfoCor 2 module on an LSM510 confocal microscope (Carl Zeiss, Jena, Germany) [15]. Illumination for FITC (GFP) and RITC was provided by continuous-wave (CW) Ar+ laser at 488 nm and He-Ne laser at 543 nm, respectively. Emission signals were collected by a 40× water-immersion objective (C-Apochromat, 40×, 1.2 NA; Carl Zeiss), split by dichroic mirror (570 nm cutoff), and filtered to green (505–530 nm) and red (600–650 nm) for FITC (GFP) and RITC, respectively, for detection by two channels of avalanche photodiodes (SPCM-200-PQ; EG&G, Quebec City, QC, Canada). Confocal pinhole diameters were set to 70 µm for FITC (GFP) and 78 µm for RITC, respectively. Fluorescence auto- and/or cross-correlation functions were calculated as previously described [37] using the ConfoCor 2 software installed in LSM510 (Ver. 4.0, Carl Zeiss, Jena, Germany). The fluorescence correlation functions *G_x_(τ)* were calculated by:

(1) $$G_{x}\left(\tau \right)=1+\frac{\langle \delta I_{i}\left(t\right)\cdot \delta I_{j}\left(t+\tau \right)\rangle }{\langle I_{i}\left(t\right)\rangle \langle I_{j}\left(t\right)\rangle }$$

where *τ* denotes the time delay; *I_i_* the fluorescence intensity of channel *i* (*r* = red, *g* = green); and *G_r_(τ)*, *G_g_(τ)*, and *G_c_(τ)* denote the auto-correlation function of the red channel (*i* = *j* = *x* = *r*), the auto-correlation function of the green channel (*i* = *j* = *x* = *g*), and the cross-correlation function (*i* = *r*, *j* = *g*, *x* = *c*), respectively.

The *G(τ)* values obtained from nanoparticles in solution were fit using a one-component model, while those obtained from cells were fit using a two-component model:

(2) $$G_{x}\left(\tau \right)=1+\frac{1}{N}\underset{i}{\sum }F_{i}{\left(1+\frac{\tau }{\tau_{i}}\right)}^{-1}{\left(1+\frac{\tau }{s^{2}\tau_{i}}\right)}^{-1/2}$$

where *F_i_* and *τ_i_* are the fraction and diffusion time of component *i, N* is the average number of fluorescent molecules/particles in the detection volume defined by the radius *w_0_* and the length *2z_0_*, and *s* is the structure parameter representing the ratio *s = z_0_/w_0_*. The structure parameter was calibrated using the known diffusion coefficient (*D*, 280 µm^2^ s^−1^) of rhodamine 6G (Rh6G) standard solution at 25°C [11,38]. All measured fluorescence auto- and cross-correlation functions (FAF and FCFs) were fitted by the software installed on the ConfoCor 2 system using this model [15,37]. For fluorescent NPs in solution, FAF and FCFs were fitted to a one-component model (*i* = 1). FAFs for NPs in living cells were fitted to a one- or two-component model (*i* = 2) with additional triplet terms to estimate the diffusion coefficient. Two-component models were used to model the NPs in the cytosol: one component represents a photo-kinetic term (i.e., fluorescence decay such as blinking) and the other component represents slow diffusion (vesicle-trapped diffusion). The mean fractional ratio (*F_i_* = 2) of diffusing NPs was indicated as a percentage. Immobilized aggregate-induced photobleaching data were excluded from the diffusion analysis. The *D* value of the nanoparticles in the solutions and cells were determined from the *D* value of Rh6G (280 µm^2^ s^−1^) and the measured diffusion times for Rh6G and fluorescent NPs. The diffusion time of component *i* is related to the diffusion coefficient *D_i_* of component *i* by:

(3) $$\tau_{i}=\frac{w_{xy}^{2}}{4D_{i}}$$

The diffusion of a spherical molecule is related to the absolute temperature, *T*, the hydrodynamic radius of the spherical molecule *r_i_*, the fluid-phase viscosity of the solvent *η*, and the Boltzmann constant *k_B_* by the Stokes–Einstein equation as follows:

(4) $$D_{i}=\frac{\kappa_{B}T}{6\pi \eta r_{i}}$$

In analysis of FCCS data, the amplitude of the cross-correlation function was normalized to the amplitude of the autocorrelation function of RITC to calculate the relative cross-correlation amplitude:

(5) $$\frac{G_{c}\left(0\right)-1}{G_{r}\left(0\right)-1}$$

### 2.6. UV-VIS Absorption Spectroscopy

Dual-color silica-based fluorescent nanoparticle (SiO_2_-NP) suspensions were prepared at 0.1 mg mL^−1^ in distilled water or PBS buffer. Following sonication, scans accumulated three times on an S-3100 UV-vis spectrophotometer (Scinco Co. Ltd., Seoul, Korea). Measurements were performed from 1100 to 200 nm through a 1 mL glass cuvette at room temperature.

## 3. Results and Discussion

**Figure 1:**
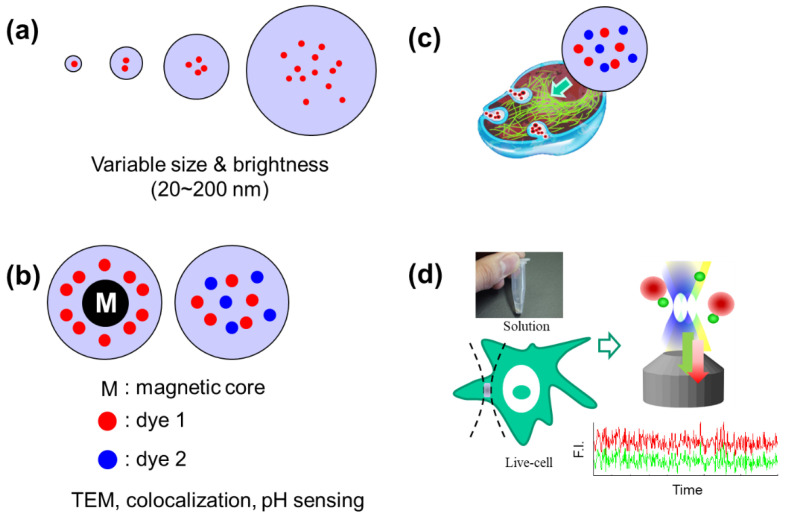
Schematic diagram depicting the variety of synthesized fluorescent nanoparticles (NPs) and the fluorescence correlation method. (**a**) Schematic representation of silica-based fluorescent NPs (SiO_2_-NPs) with different diameters and brightnesses. (**b**) Schematic representation of a silica-based fluorescent NPs with magnetic core (CoFe_2_O_4_/SiO_2_-NP) and dual-colored SiO_2_-NPs. (**c**) Schematic representation showing the various NPs used as analytical probes or sensors during the cellular uptake process. (**d**) Schematic representation of fluorescence correlation methods (i.e., FCS and FCCS) in aqueous solution sample and in live cells.

Application of multiple analytical and imaging modalities to quantitatively detect single NPs would provide valuable information when the cellular status of NPs uptaken by cells is spatio-temporally investigated. For complementary analysis of NPs using TEM, DLS, and CLSM based FCS/FCCS, various silica-based fluorescent NPs containing RITC and/or FITC were designed (Figure 1, see also Table 1). In addition, fluorescent NPs with a cobalt ferrite CoFe_2_O_4_ core and dual-colored NPs were designed for high contrast between the shell and core structure in TEM. Finally, as depicted in Figure 1, if well-defined NPs with various sizes and fluorescence characteristics can be synthesized, and the characteristics of these NPs can be defined with complementary analytical and imaging methods, then the cellular analyses can be linked by FCS/FCCS.

### 3.1. Characteriation of Synthesized Nanoparticles

**Figure 2:**
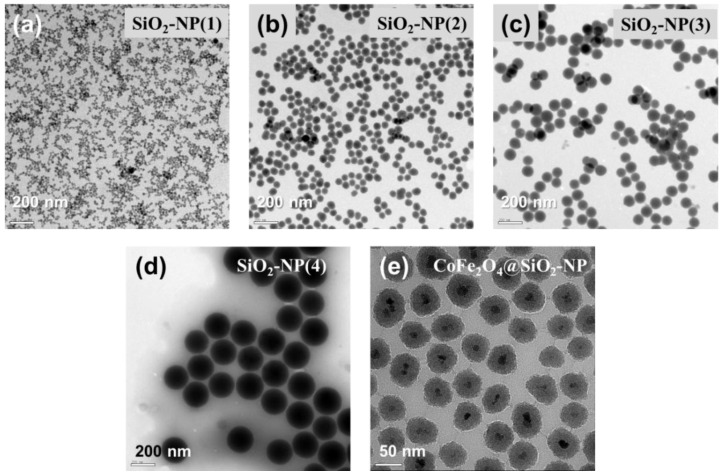
Size and morphology of silica-based nanoparticles. (**a**–**d**) Electron micrographs of fluorescent SiO_2_-NPs with sizes varying from 20 to 200 nm in diameter: (**a**) 22 nm diameter, (**b**) 56 nm diameter, (**c**) 97 nm diameter, (**d**) 230 nm diameter. Scale bars, 200 nm. (**e**) Electron micrograph of fluorescent CoFe_2_O_4_/SiO_2_-NP of 60 nm diameter shows consistent dispersal, shape, and size. Scale bar, 50 nm.

Highly reproducible and precise synthesis of nanoparticles with homogenous size and morphology is essential for their application to the study of intracellular dynamics. Four SiO_2_-NPs with different sizes were fabricated and then imaged by TEM for determination of the consistency and range of accessible sizes. As shown in Figure 2, the synthesized SiO_2_-NPs were highly spherical in structure and varied in size from 22 to 230 nm (Table 1). CoFe_2_O_4_/SiO_2_-NPs were also successfully synthesized and showed a clear core/shell structure. These CoFe_2_O_4_/SiO_2_-NPs were also highly spherical in structure and monodisperse. In TEM, the CoFe_2_O_4_/SiO_2_-NPs had visibly higher contrast at the center of core and diameters of 50~60 nm. Due to the increased contrast, the ferrite core can be clearly differentiated from the silica shell structure in EM imaging.

**Figure 3:**
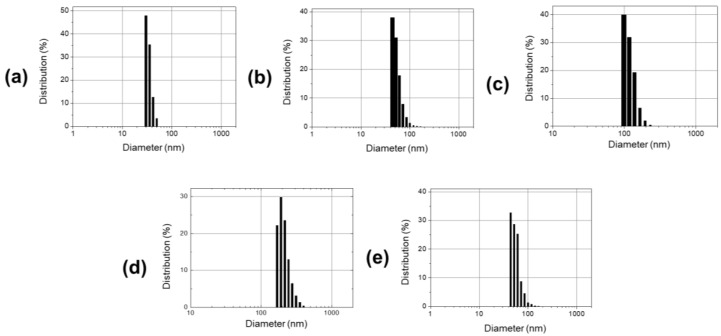
Size characterizations of synthesized SiO_2_-NP and CoFe2O4/SiO2-NP by DLS analysis in aqueous solution. (**a**–**d**) Diameter distributions of synthesized SiO_2_-NP, matching Figure 2a–d. (**e**) The intensity-size distribution graph of the cobalt ferrite nanoparticles, matching Figure 2e.

In addition to TEM analysis with dried nanoparticles, hydrodynamic diameters of the nanoparticles in aqueous solution were also quantified by DLS (Figure 3). Size distributions based on intensity are ideal for determining hydrodynamic diameter [22,39] and are plotted in Figure 3 for all nanoparticles shown in Figure 2a–d. Diameters followed a log-normal distribution. The smallest-sized nanoparticles were distributed around 28.8 ± 5.3 nm, while the largest were around 210.7 ± 15.2 nm in hydrodynamic size when measured by DLS. The cobalt ferrite-core silica NPs shown in Figure 2e and Figure 3e had hydrodynamic diameters of 62.1 ± 3.2 nm and were thus comparable in size to the silica nanoparticles shown in Figure 2b and Figure 3b.

### 3.2. Fluorescence Correlation Analysis

**Figure 4:**
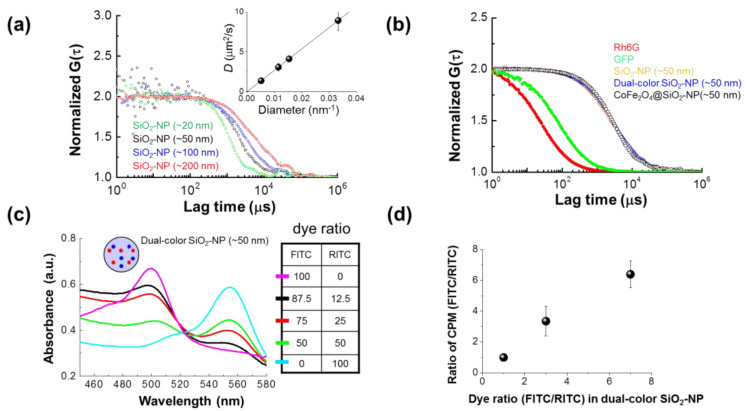
Characterization of SiO_2_ fluorescent NP and dual color SiO_2_ NP (diameter ~60 nm) using FCS and dual-color FCCS. (**a**) Representative normalized auto-correlation functions (*G*(*τ*)s) of the fluorescent NPs having diameters ranging from 20 to 200 nm are shown. Inset, a plot of diffusion coefficient versus hydrodynamic diameter is shown for each nanoparticle in distilled water. (**b**) Normalized autocorrelation functions *G(τ)* are compared between rhodamine 6G (Rh6G), recombinant GFP molecule, and the synthesized fluorescent NPs. (**c**) Absorption spectra of dual-color SiO_2_ NP shows the dependence of the dual-color SiO_2_ NP absorption spectrum upon the molar ratio of FITC and RITC dye at the time of fabrication. Insert shows corresponding molar dye ratios. (**d**) The ratio of brightness (count per molecule; CPM in kHz) of dual color SiO_2_ NP, evaluated by FCS, depends on the molar ratio of the two dyes. (See also Table 2).

The hydrodynamic sizes of the CoFe_2_O_4_/SiO_2_ nanoparticles were also obtained from fluorescence auto-correlation functions of SiO_2_-NP in distilled water or PBS buffer. Evaluated hydrodynamic sizes in distilled water are shown in Figure 4a. The obtained diffusion coefficients, *D,* are visibly linear with the reciprocal of nanoparticle diameter, demonstrating the applicability of the Stokes–Einstein relation, and a linear fit to *D/(2r)* in distilled water had a slope of 0.2676 ± 0.0002 µm^3^ s^−1^, demonstrating that the NPs are monodisperse in aqueous solution and consistent with the result from TEM and DLS analysis (Table 1). In Figure 4b, it can be seen that the observed diffusion is slow compared to the much smaller molecules of standard fluorophores Rh6G (~1 nm) and synthesized GFP (~3.5 nm), while the ferrite core-shell fluorescent nanoparticle maintains almost identical diffusion rates to the equivalently-sized silica-based fluorescent and dual-color fluorescent nanoparticles. For FCS analysis, the hydrodynamic sizes of the nanoparticles increase with particle size, from 30 ± 3.1 nm for the smallest nanoparticles to 200 ± 12.4 nm for the largest. CoFe_2_O_4_/SiO_2_ nanoparticles have a statistically irrelevant hydrodynamic size difference from the nearest-sized silica nanoparticle, at 65 ± 3.5 nm (vs. silica 63 ± 2.5 nm). The zeta potential is also an important property of nanoparticles, since the electric potential of colloidal suspensions is highly relevant for nanoparticle interactions with the cell surface. The derived zeta potentials, as summarized in Table 1, are increasingly negative with particle size, ranging from −31.0 ± 5.8 mV for the smallest nanoparticles to −51.1 ± 0.7 mV for the largest nanoparticles. Incorporation of cobalt ferrite cores has no measurable effect on the zeta potential or fluorescence.

The surface potentials (Table 1) show monotonically trending behaviors with particle size, which suggests a relationship between the surface charge and the accumulative chemical synthesis process. A similar relation for zeta potential has been previously attributed to the chemistry of the Stöber synthesis process used for the NPs in this paper [40]. For volumetric reasons, the brightness per particle was also observed to increase monotonically with particle size (Table 2).

A number of dual-color fluorescent shell silica nanoparticles (dual-color SiO_2_-NP) were fabricated, with FITC:RITC ratios ranging from 100:0 to 0:100. Absorption spectra demonstrating the effect of dual doping are shown in Figure 4c. Increasing relative concentrations of FITC are associated with an absorption peak at 500 ± 3 nm, while increasing relative concentrations of RITC are associated with an absorption peak at 554 ± 2 nm. In RITC, a slight blue-shift of the absorption peak (less than 10 nm total) is visible at the lowest concentrations.

In comparison with FCS, dual-color FCCS is more robust to background noise, and thus, offers advantages for analysis in the cell. The cross-correlation analysis, however, is sensitive to relative fluorescence intensities. The ratios of fluorescence intensities in CPM for FITC vs. CPM for RITC were plotted in Figure 4d for the dual-color SiO_2_-NP as a function of the dye ratios, revealing that the brightness ratio is closely correlated with the doping ratio at these concentrations. Dual-color SiO_2_-NP with a 1:1 FITC:RITC dye ratio was found to be ideal for FCCS.

**Figure 5:**
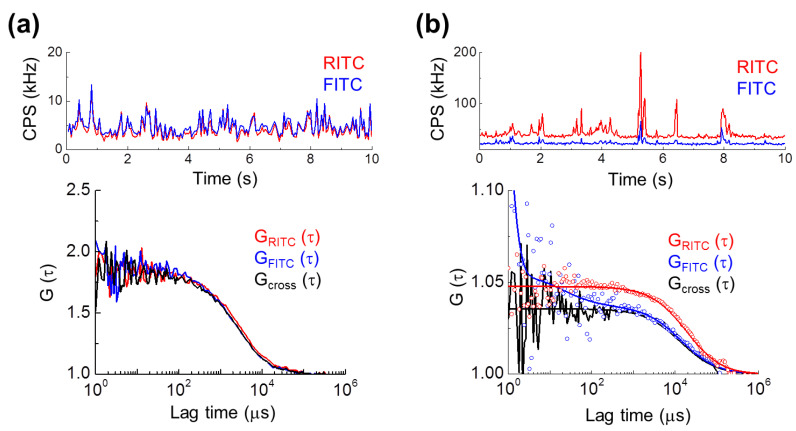
FCCS analysis of dual-color SiO_2_-NP in aqueous solution and in a single live HeLa cell. SiO_2_-NPs having ~60 nm diameter and 1:1 FITC:RITC dye ratio were analyzed by FCCS. (**a**) Representative spatially-averaged fluorescence intensity of the nanoparticles is shown over a 10 s interval (upper). Fluorescence intensity is presented as counts per second (CPS) in kHz. Two representative fluorescence auto-correlation functions (*G(τ)*, blue and red) and the corresponding dual-color fluorescence cross-correlation function (black) measured in PBS buffer are shown (lower). (**b**) Representative spatially-averaged fluorescence intensity of the nanoparticles in the cytosol of a live Hela cell after medium incubation is shown (upper). Two representative fluorescence auto-correlation functions (blue and red) and the dual-color fluorescence cross-correlation function (black) are shown as measured in live Hela cells after media incubation of 1 h (lower).

As can be observed in Figure 5, fluorescence intensities of dual-color SiO_2_-NP detected by FCCS were roughly equal for both the FITC and RITC channels in PBS, but RITC was observed to have roughly two- to four-fold intensity when the NPs were up-taken in the living cell. This relative decrease in the fluorescence intensity of FITC is likely to be the result of decreasing FITC fluorescence due to pH effects as NPs are trapped in acidic cellular endosomes [41,42,43]. The two fluorescence auto-correlation functions were closely correlated both to each other and to the cross-correlation function in PBS, and *G(τ)* fit best to a one-parameter free dispersion model, indicating free diffusion of NPs. This result demonstrated that the dyes are completely confined within the NPs and no free dyes are detected. Furthermore, the dyes are also confined within the nanoparticles for cell analysis, even though the relative fluorescence intensity of FITC compared to RITC is decreased, demonstrating that dual-color SiO_2_-NP with very high brightness is useful as standard probe for cell analysis.

In our previous study using FCS [15], it was found that the diffusion of silica nanoparticles was significantly slowed in the intracellular environment compared to in solution. The FCCS result shown in Figure 5 is consistent with this previous result.

### 3.3. Comparison between D Values and Spatial Distributuion during Cellular Uptake

**Figure 6:**
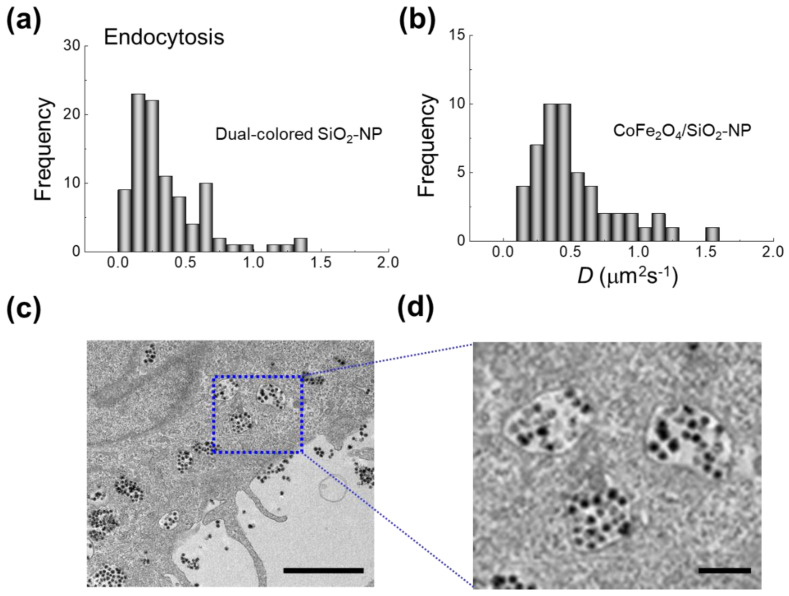
Cellular uptake and diffusional dynamics of fluorescent CoFe_2_O_4_/SiO_2_-NP. Distribution of diffusion coefficients of (**a**) dual-color SiO_2_-NP and (**b**) CoFe_2_O_4_/SiO_2_-NP after cellular uptake, as evaluated by FCS and FCCS. (**c**) Electron micrograph demonstrating fluorescent CoFe_2_O_4_/SiO_2_ uptake in a HeLa cell, showing the wide variety of endocytosis statuses. Scale bar 2 µm. (**d**) Blow up image of the boxed region, as CoFe_2_O_4_/SiO_2_-NPs are incorporated into endosomes. Scale bar 500 nm.

When analyzed over whole cells using LCSM-based FCS, the diffusion coefficients of dual-color SiO_2_-NP and CoFe_2_O_4_/SiO_2_-NP in live HeLa cells were observed to be widely divergent, which is explained by captured electron micrographs. The distribution of the diffusion coefficients of the two types of NPs in the cell was almost similar. As can be seen in Figure 6, roughly 70% of observed NPs were taken into the cell by endocytosis, while 30% remained in culture media. Due to the variety of NP fates, the distribution of diffusion coefficients *D* was observed to be multimodal, with *D* varying from 0.01 to 1.5 µm^2^ s^−1^. Previous studies have shown a similar diffusivity of 50-nm-diameter silica NPs in HeLa cells [15]. It is likely that the low end of the observed range reflects large groups of NPs trapped in single endosomes, and thus agglomerated, whereas the high end of the observed range reflects single NPs free in the cytosol. Since the majority of NPs agglomerate in medium-sized endosomes, a modal diffusion coefficient *D* of 0.2~0.25 µm^2^ s^−1^ was observed. This result agrees well with the results of a previous study [15].

While numerous studies have shown that the interaction of nanomaterials with parts of cells, such as the lipid bilayer of the cellular membrane, is critical in many applications such as phototherapy, imaging, and drug/gene delivery [44], it remains challenging to quantitatively analyze the dynamic behavior and interactions of various nanomaterials and to apply comparable analytical methods both to samples in solution and to those uptaken in cells. Previously, it has been shown that silica-based fluorescent nanoparticles without specific surface modification are well-suited for cell imaging analyses due to their biological inertness, low intracellular interactivity, and easy uptake by endocytosis or by electroporation [15,45,46]. Therefore, if silica-based nanoparticles having various physicochemical properties can be synthesized and the corresponding properties such as size, brightness, and photostability, can be quantitatively analyzed at the level of single particles both in aqueous solutions and living cells, it will be helpful in evaluating other nanomaterials and systematically understanding cellular uptake process.

In the case of dual-color fluorescent nanoparticles, it was shown that the ratio and brightness of each fluorescence can be quantified using the FCCS technique, and that changes in the environment surrounding the NPs are expressed through the changes in the relative fluorescence brightness. In addition, it was proven that by applying nanoparticles containing two fluorescent molecules and FCCS analysis, information can be obtained not only about the diffusional behavior of intracellular nanoparticles (i.e., effective cellular viscosity and interactions with cellular organelles), but also about the interactions between the two fluorescent dyes. Nevertheless, since FCS and FCCS are techniques that detect only a small part of a cell, it is preferable to analyze in parallel the distribution and aggregation state across the entire cell using a confocal microscope or an electron microscope, as demonstrated by the use of fluorescent CoFe_2_O_4_/SiO_2_-NP. As shown in this study, a more rational interpretation of the diffusion coefficient distribution becomes possible when FCS and EM analysis are linked.

## 4. Conclusions

The standardization and evaluation of NPs with well-defined physicochemical properties is an important step in the characterization of nanoparticle entry, diffusion, and interaction within the living cell as well as in aqueous solution. In this study, we have successfully demonstrated that highly soluble SiO_2_- and CoFe_2_O_4_/SiO_2_-NPs containing fluorescent dyes with enhanced brightness and multiple colors are useful as a standard probe for multiple imaging modalities when combined with analytical FCS and FCCS methods. The well-defined physicochemical properties of SiO_2_-NPs and their low-aggregation property in aqueous solution make these NPs especially appropriate for the quantification of diffusion dynamics in living cells. Moreover, FCS and FCCS can provide detailed information about the cellular microenvironments surrounding the various silica-based nanoparticle probes. Our method will be helpful when evaluating nanoparticle- and nanocarrier-based drug delivery at the sub-cellular level.

## Figures and Tables

**Table 1:** Summary of average values of nanoparticle diameter and zeta potential.

Measurement	SiO_2_-NP(1)	SiO_2_-NP(2)	SiO_2_-NP(3)	SiO_2_-NP(4)	CoFe_2_O_4_/SiO_2_
TEM diam. (nm) ^1^	22.2 ± 1.8	56.1 ± 4.2	97.1 ± 4.4	226.6 ± 13.9	60.0 ± 4.2
DLS diam. (nm) ^1^	28.8 ± 5.3	57.8 ± 3.2	117.3 ± 2.4	210.7 ± 15.2	62.1 ± 3.2
FCS diam. (nm) ^1^	30 ± 3.1	63 ± 2.5	91 ± 5.1	200 ± 12.4	65 ± 3.5
Zeta (mV)	−31.0 ± 5.8	−34.1 ± 2.0	−46.8 ± 0.9	−51.1 ± 0.7	−32.1 ± 4.0

^1^ TEM diameter is the diameter of dry nanoparticles in nm, while DLS and FCS reflect the hydrodynamic diameter in distilled water in nm.

**Table 2:** Summary of brightness of synthesized silica-based nanoparticles.

Measurement	SiO_2_-NP(1)	SiO_2_-NP(2)	SiO_2_-NP(3)	SiO_2_-NP(4)	CoFe_2_O_4_/SiO_2_
CPM (kHz) ^1^	40.1 ± 11.6	45.4 ± 6.2	201 ± 53	784 ± 286	45.4 ± 6.2

^1^ CPM represents the averaged fluorescent intensity per single nanoparticle evaluated by FCS with RITC channel.

## Data Availability

Data is contained within the article.
